# Fabrication of a Microfluidic Test Device with a 3D Printer and Its Combination with the Loop Mediated Isothermal Amplification Method to Detect *Streptococcus pyogenes*

**DOI:** 10.3390/mi15030365

**Published:** 2024-03-07

**Authors:** Hayriye Kirkoyun Uysal, Meltem Eryildiz, Mehmet Demirci

**Affiliations:** 1 Department of Medical Microbiology, Istanbul Faculty of Medicine, Istanbul University, Istanbul 34093, Turkey; hkirkoyun@yahoo.com; 2 Department of Mechanical Engineering, Faculty of Engineering and Architecture, Istanbul Beykent University, Istanbul 34396, Turkey; meltem87@gmail.com; 3 Department of Medical Microbiology, Faculty of Medicine, Kirklareli University, Kirklareli 39100, Turkey

**Keywords:** *S. pyogenes*, LAMP, microfluidic device, 3D printer

## Abstract

New rapid, reliable, and cost-effective alternative systems are needed for the rapid diagnosis of *Streptococcus pyogenes*. The aim of this study was to fabricate a microfluidic test device to detect *Streptococcus pyogenes* by combining the Loop-mediated isothermal amplification method via a 3D printer. Microfluidic test devices were designed in CATIA V5 Release 16 software, and data were directly transferred to a 3D printer and produced using the FDM method with biocompatible PLA filament. The *S. pyogenes* ATCC 19615 and different ATCC strains was used. Following identification by classical culture methods, a 0.5 McFarland suspension was prepared from the colonies, and DNA isolation was performed from this liquid by a boiling method. *S. pyogenes* specific *speB* gene was used to desing LAMP primer sets in PrimerExplorer V5 software and tested on a microfluidic device. LAMP reactions were performed on microfluidic device and on a microcentrifuge tube separately. Both results were analyzed using the culture method as the standard method to diagnostic values. Melting curve analysis of the amplicons of the LAMP reactions performed on a LightCycler 480 system to detect amplification. Among the 50 positive and 100 negative samples, only four samples were found to be false negative by LAMP reaction in a microcentrifuge tube, while eight samples were found to be false negative by LAMP reaction on a microfluidic device. Six samples were found to be false positive by the LAMP reaction in the microcentrifuge tube, while ten samples were found to be false positive by the LAMP reaction on a microfluidic chip. The sensitivity, specificity, positive predictive value, and negative predictive value of the LAMP reactions performed in the microcentrifuge tube and on the microfluidic device were 92–84%, 94–90%, 88.46–80.77%, and 95.92–91.84%, respectively. The limit of detection (LOD) was found to be the same as 1.5 × 10^2^ CFU/mL and the limit of quantification (LOQ) values of the LAMP reactions were performed on the microcentrifuge tube and on the microfluidic device were 2.46 × 10^2^–7.4 × 10^2^ CFU/mL, respectively. Cohen’s kappa (κ) values of the LAMP reactions were performed on the microcentrifuge tube and on the microfluidic device were 0.620–0.705, respectively. In conclusion, our data showed that the LAMP method can be combined with microfluidic test device to detect *S. pyogenes*, this microfluidic device can be manufactured using 3D printers and results are close to gold standard methods. These devices can be combined with LAMP reactions to detect different pathogens where resources are limited and rapid results are required.

## 1. Introduction

*Streptococcus pyogenes* (*S. pyogenes*) are Gram-positive, cocci-shaped, beta-hemolytic bacteria and can grow on enriched media [1]. *S. pyogenes,* also defined as Group A Streptococci (GAS) according to the carbohydrate antigens on the cell wall by the Lancefield Classification System, are considered important pathogens that can cause serious infections in humans [1,2]. They are also identified as causative agents in infections such as acute pharyngitis, scarlet fever, impetigo, cellulitis, necrotizing fasciitis, streptococcal toxic shock syndrome, and when not treated accurately, important post-streptococcal infections such as acute rheumatic fever and post-streptococcal glomerulonephritis. *S. pyogenes* are detected as the causative agent in 5–15% of adults and 20–30% of children who apply to the hospital every year with the complaint of sore throat, which is one of the most important reasons for hospitalizations on a global scale. Following the infection, they can cause approximately two million new cases and 500 thousand deaths every year [3,4]. *S. pyogenes* have the highest incidence rate; especially in children aged 5–15 years, and was identified as a dangerous pathogen in school-age children. *S. pyogenes* can frequently colonize the pharynx of asymptomatic individuals, especially in children, and the rate of asymptomatic *S. pyogenes* carriage is predicted to be 15–20% [5]. Failure to treat pharyngitis caused by Group A Streptococci [GAS] correctly was associated with the etiopathogenesis of rheumatic fever and rheumatic heart disease. Primary prevention from such infections is mediated by the elimination of GAS or *S. pyogenes* carriage by screening for *S. pyogenes* following an active sore throat and treatment of pharyngitis with oral antibiotics when the agent is detected [6]. Clinical symptoms of Streptococcal pharyngitis are similar to those of viral pharyngitis and nonstreptococcal pharyngitis. As a result, clinicians must use laboratory tests to confirm or exclude GAS, make appropriate treatment decisions, and avoid unnecessary antibiotic use [4]. Empirical antibiotics can be used because of the weak antibiotic resistance ability of *S. pyogenes*, but the treatment failure rate with penicillin has increased in recent years and reached 40% [7]. Timely drug therapy may control the development of inflammation and prevent the spread of pathogenic bacteria. For this reason, early detection and identification of pathogens is especially important. Traditional methods used to detect *S. pyogenes* are mainly classical culture method, immunological methods, and Polymerase Chain Reaction (PCR) [2]. The Loop-Mediated Isothermal Amplification (LAMP) method was developed in 2000 as a faster target nucleic acid amplification method to can be used reliably without the need for experienced staff and special devices, such as PCR, Real-Time PCR, and sequencing methods [2,8]. Because of these characteristics, the LAMP method can form the basis of Point-of-Care (POC) diagnostic kit systems and can be used with strain designs for many pathogens [9]. Microfluidic devices are miniaturized devices and contain channels and chambers with scale dimensions of 1 mm or less to control the flow of small-volume liquids and the technology used for their production. Combining molecular techniques with these devices may become an important option for performing Point-of-Care diagnostic testing at points where access to healthcare is limited or where there are limited resources [10]. Although traditional microfluidic manufacturing methods (e.g., soft lithography, micro-processing, embossing, and injection molding) were proven to be successful and reliable in molecular diagnostics. However, their use still remains limited because of reasons such as the need for molding, the inability to perform rapid prototyping, the need for large quantities of production, the need for serious expertise, and high costs [11,12,13]. Desktop 3D printing systems (e.g., fused deposition modeling (FDM) and SLA (Stereolithography))*,* which have become very popular in recent years, are accessible, low-cost, and easy-to-use devices that require less expertise. For this reason, they are increasingly used in prototyping, customizing, and manufacturing microfluidic devices for diagnosis and research [14].

Three-dimensional printing technology offers an important opportunity to the developed point of care diagnostic systems, to detected drug toxicity or to created organoids [15,16,17,18]. The researchers detected an inflammatory biomarker for the diagnosis of sepsis with the microfluidic chip design they designed with a three-dimensional printer and reported good performance in terms of sensitivity, specificity, reproducibility, and stability [15]. In addition, researchers have created diagnostic platforms with high analytical sensitivity by integrating fluorescent probes into point of care diagnostic systems integrated with microfluidic chips and have shown that this can be useful for diagnostic tests [16]. Three dimensional printing enables accessible organ-on-chip devices for multicellular spheroid cultures, mimicking cell interactions, and facilitating drug/toxicity studies [17]. Using this technology, the researchers have also developed a new method using 3D printed microfluidic chips to create organoids with blood vessels that realistically grow and interact with the organoid. This method uses human stem cells and specialized chips to create small channels for blood vessels to grow alongside the organoid [18].

Combining the Loop-Mediated Isothermal Amplification (LAMP) method with microfluidic devices will be an important step for the development of point-of-care systems that can be used for rapid diagnostic biomarkers [19]. This study was aimed to create and manufacture a microfluidic test device prototype by using an FDM-mediated 3D printer to detect *Streptococcus* pyogenes by combining the Loop-Mediated Isothermal Amplification (LAMP) method.

## 2. Materials and Methods

### 2.1. Microfluidic Test Device Design with 3D Printer

**Figure 1:**
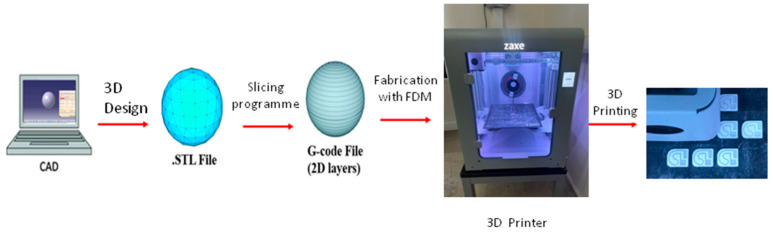
Microfluidic device fabrication steps.

The fused deposition modeling (FDM) method was chosen to produce the microfluidic device. Easy and fast manufacturing was achieved with this method. Also, there was no need to manufacture an extra mold as in Injection Molding and Soft Lithography Methods, and the microfluidic test devices were designed by using the CATIA V5 Release 16 Program. The microfluidic device dimensions were 15 mm × 12.5 mm × 2.4 mm, the channel dimension was 500 µm × 500 µm, and the channel length was 41,750 µm. For this specific microfluidic device with a 25 µL total chamber volume, the required volume of the LAMP mixture is approximately 14.6 µL. The reaction withdrawal chamber enables easy and non-contaminating extraction of the amplified product for analysis, while the reaction chamber houses the LAMP reaction itself. Spiral microfluidic channels are chosen for their ability to the compact design of for incorporating long channels within a small footprint, enabling high-throughput processing of fluids. This is advantageous for applications requiring rapid analysis of large sample volumes [20]. The microfluidic devices were sliced and converted into G-codes in the xDesktop 2.1.6 Program and were manufactured by using the Z1 Plus Model FDM 3D Printer (Zaxe, Istanbul, Turkey). Biocompatible polylactic acid (PLA) filament, which is frequently preferred in tissue engineering and the production of microfluidic disposable devices in recent years, was used as the material in microfluidic device manufacturing [21]. To perform this production, the 3D printer Zaxe was procured from Turkey for the production of 1.75 mm diameter polylactic acid (PLA) filament microfluidic devices (Figure 1).

Table 1 shows the printing parameters used in microfluidic device production. Individual chips were enclosed in airtight packaging, and subsequently stored in a controlled environment with low humidity, avoiding direct sunlight and extreme temperatures.

**Figure 2:**
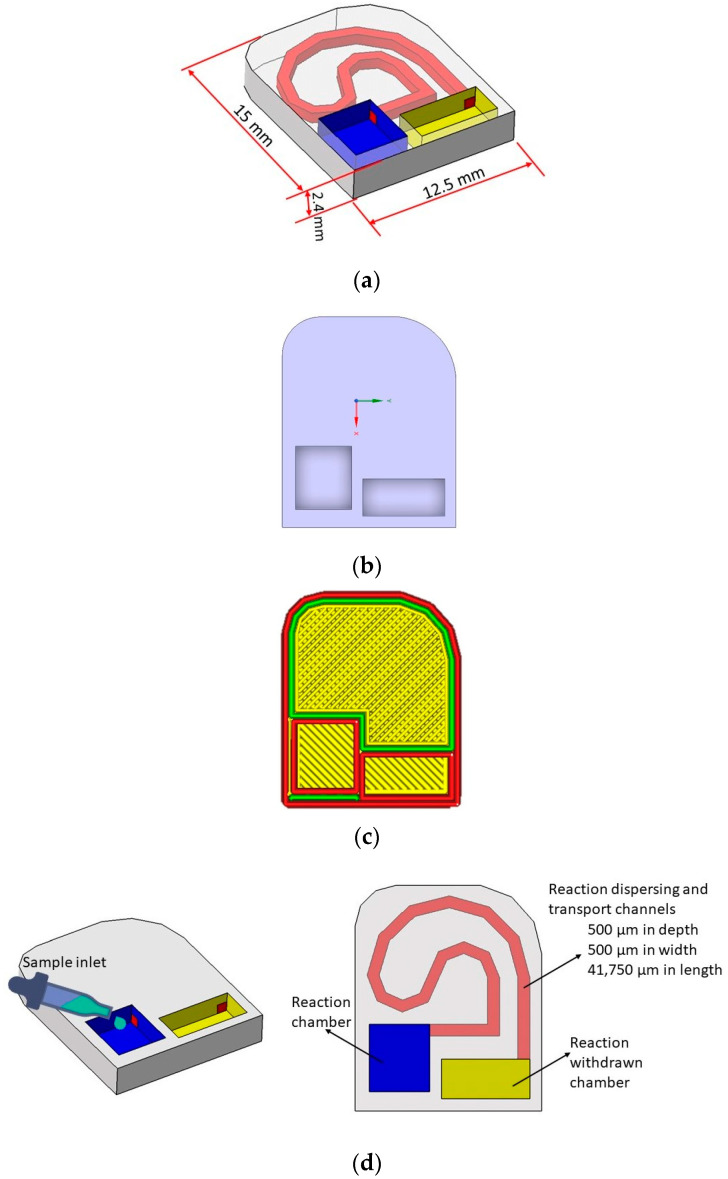
(**a**) 3D printing parameters of Microfluidic device; (**b**) CAD design of Microfluidic device; (**c**) Sliced model of Microfluidic device; (**d**) Self-driven microfluidic device production characteristics.

The drawings associated with the design are given in Figure 2a–d.

**Figure 3:**
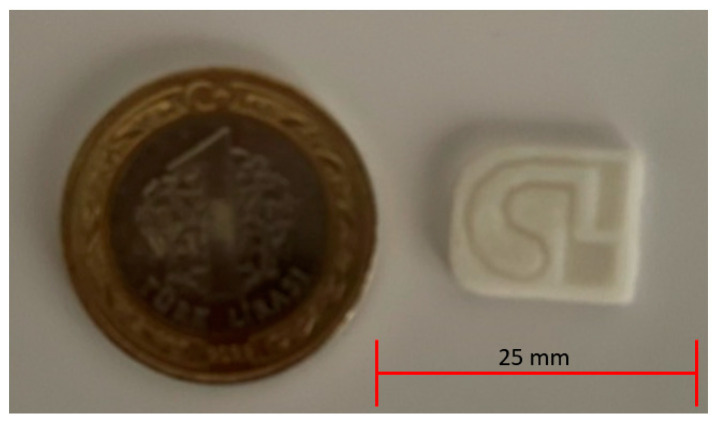
Microfluidic device design and its comparison with a coin.

The microfluidic test device that was produced with a 3D printer and its size comparison with a coin are given in Figure 3.

### 2.2. Production and Identification of S. pyogenes ATCC Strain

*S. pyogenes* the American-Type Culture Collection (ATCC) 19615 was used as a positive control. The strain was culture classically by using a 5% sheep blood agar medium. Identification was performed by detecting classical biochemical characteristics such as Gram-staining and catalase. 

### 2.3. DNA Isolation from S. pyogenes Colonies

*S. pyogenes* ATCC 19615 colonies (1.5 × 10^6^ CFU/mL) diluted to 0.5 McFarland density were used and DNA isolation was performed with the Bacterial Boiling Method. Standard concentrations were obtained by using 10-fold dilutions of the obtained DNA, which were then used as standards in optimization.

### 2.4. LAMP PCR Primer Set Design

To create a microfluidic *S. pyogenes* detection device based on the Loop-Mediated Isothermal Amplification (LAMP) method, following device production, LAMP Primer Sets were designed in PrimerExplorer Ver.5 software by using FASTA sequences of the *Streptococcus pyogenes*-specific *speB* gene (Gene Bank Accession Number: M86905.1; http://primerexplorer.jp/lampv5e/index.html) (accessed on 4 March 2024). Forward and reverse outer primers (F3 and B3), forward and backward inner primers (FIP and BIP) were detected with online software and these primers were supplied. To target the single-stranded loop regions within the B1/B2 or F1/F2 sequences, Loop Primers were generated using the “pick primer” function in NCBI’s backward loop primer (LB) or Forward loop primer (LF) databases. These primers share complementary sequences with the desired loop regions. Specific primer sequences obtained following FASTA sequence loading for LAMP design are provided in Table 2. PrimerExplorer software guidelines were followed to select suitable primers among different primer sequences and selections were made.

### 2.5. Conducting LAMP Study on the Microfluidic Device and on a Microcentrifuge Tube

The LAMP reaction was performed in two separate amplifications in the microfluidic device and the microcentrifuge tube. Manufacturer instructions were followed for LAMP protocols. The total volume of each reaction was 25 μL (including 4 μL of nucleic acid in the 21 μL LAMP PCR Mixture) and Bsm DNA Polymerase (Thermo Scientific, Waltham, MA, USA) (8 U/μL) was used according to the manufacturer’s instructions. The final concentration was 0.8 μM for FIP and BIP, 0.2 μM for F3 and B3, and 0.2 µM LF and LB in the reactions. 

A reaction setup was designed for per reaction as 4 µL of 25 mM MgCl_2_, 3.5 µL of 10 mM dNTP mix, 1 µL of each FIP and BIP, 0.5 µL of each F3 and B3, 2.5 µL of 10× Bsm Buffer, 1 µL of each LoopF/B primers, 1 µL of 8 U/µL Bsm DNA Polymerase, 2 µL of Nuclease-free water

**Figure 4:**
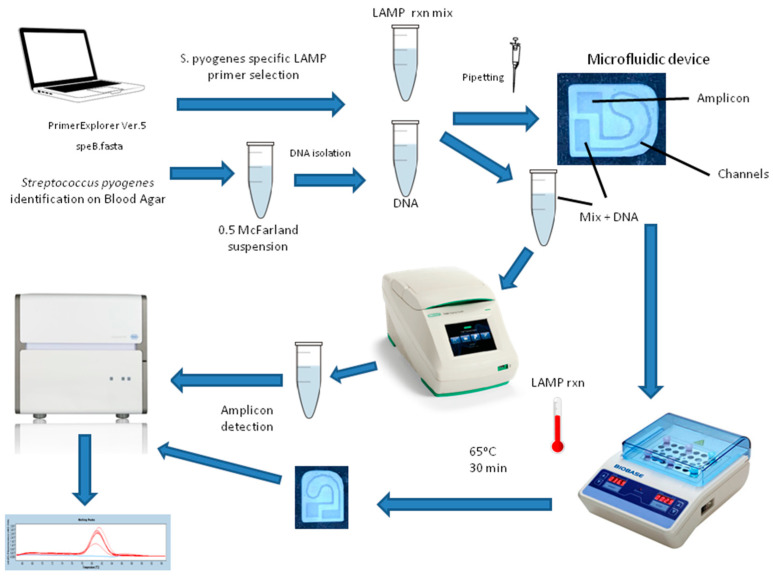
LAMP protocol steps on the microfluidic device and on the microcentrifuge tube.

LAMP Protocols were used for each microcentrifuge tube with the T1 PCR System (Bio-Rad, Hercules, CA, USA). A dry heat block was used for LAMP in microfluidic devices. On the microfluidic device and on the microcentrifuge tube, the LAMP Protocol was applied similarly for 30 min at 65 °C. Amplification products were checked on the LightCycler 480 (Roche Diagnostics, Mannheim, Germany) Real-Time PCR via melting curve protocol. Melting analysis and melting curve graphs were used to evaluate the LAMP reactions. LightCycler 480 sealing foil (Roche Diagnostics, Mannheim, Germany), also used in qPCR reactions, was used to prevent evaporation during LAMP reactions on microfluidic devices. All LAMP protocol steps on the microfluidic device and on the microcentrifuge tube were summarized in Figure 4.

The protocol optimized with *S. pyogenes* and the standard DNAs of different ATCC strains from our collection were used both LAMP Reactions performed on the microcentrifuge tube and performed on the microfluidic device.

In LAMP reactions, the *S. pyogenes* ATCC 19615 strain was used as a positive control in the analysis of the results. A total of 50 positive samples were studied with 10 repetitions of each dilution standard for positive control (in the range of 10^6^–10^2^ CFU/mL). For negative control, *Streptococcus agalactiae* ATCC 12386, *Streptococcus mutans* ATCC 25175, *Haemophilus influenzae* ATCC 49247, *Staphylococcus epidermidis* ATCC 12228, *Lactobacillus acidophilus* ATCC 4356, *C. albicans* ATCC 10231, *Klebsiella pneumoniae* ATCC 13883, *Staphylococcus aureus* ATCC 29213, *Escherichia coli* ATCC 25922, and *Pseudomonas aeruginosa* ATCC 27853 standard strains were used. For each isolate, a total of 10 replicates of the LAMP study were performed.

### 2.6. The Test Performance Indicators Calculations

The test performance indicators calculations were made for LAMP results by considering the results of the classical culture method. True positive/(true positive + false negative) formula was used for sensitivity measurement, the true negative/(true negative + false positive) formula was used for specificity measurement, the true positive/(true positive + false positive) formula was used for positive predictive value measurement, and the true negative/(true negative + false negative) formula was used for negative predictive value measurement [22]. The Beer-Lambert Law was used in the limit of detection (LOD) and limit of quantification (LOQ) calculations [23].

The Cohen’s Kappa (κ) value was calculated as an indicator of the compatibility with the culture results of the LAMP reaction that was performed on the microcentrifuge tube and the performed on the microfluidic device. The agreement between the results was evaluated by using the κ coefficient. It was evaluated that κ values of 0.40 and below indicated weak correlation, κ values between 0.41 and 0.60 indicated good fit and κ values above 0.60 indicated strong fit. For assessing inter-rater reliability in nominal data with two or more unordered categories, The Cohen’s Kappa (κ) value provides a robust measure of agreement, accounting for chance agreement [24].

## 3. Results

The positive and negative controls used in the study are shown in Table 3.

LAMP reactions were performed on the microfluidic device and on a separate microcentrifuge tube in the study. Taking the classical culture method as the gold standard method, the results of the LAMP reactions performed on the microfluidic device and microcentrifuge tube were analyzed in this context.

Investigating the optimal conditions for LAMP amplification revealed a sweet spot of 65 °C, where the reaction achieved its peak performance. Interestingly, a range of temperatures between 61 °C and 67 °C yielded statistically indistinguishable results, demonstrating the assay’s robustness within this window. Pushing the temperature beyond 67 °C, however, proved detrimental, causing the LAMP reaction to grind to a halt. In terms of reaction time, while 30 min delivered the most efficient amplification, the process exhibited commendable stability throughout the tested range, successfully reaching completion even after 60 min. This extended window of tolerance for both temperature and time signifies the flexibility and reliability of the LAMP assay for practical applications.

Among the 50 positive samples and 100 negative samples that were determined to be *S. pyogenes*-positive by the culture method, false negative results were detected in only four samples with the LAMP Reaction that was applied in the microcentrifuge tube, and eight samples were determined to be false negative in the LAMP reaction applied on the microfluidic device. False-negative samples in both the LAMP Reactions performed on the microcentrifuge tube and on the microfluidic device were detected in the sample of the *S. pyogenes* ATCC standard that had a concentration of 10^2^. Although false positive results were detected in six samples with the LAMP reaction applied on the microcentrifuge tube, ten samples were found to be false positive in the LAMP reaction applied on the microfluidic device (Table 4a,b).

The results of the test performance indicators that were calculated according to positivity and negativity are given in Table 5. The sensitivity, specificity, positive predictive value, and negative predictive value of the LAMP reactions that were performed on the microcentrifuge tube and on the microfluidic device were found to be 92–84%, 94–90%, 88.46–80.77%, and 95.92–91.84%. For example, the formula 46/(46 + 4) was used for sensitivity determination and the formula 94/(6 + 94) was used for specificity determination of the LAMP rxn on microcentrifuge tube.

**Figure 5:**
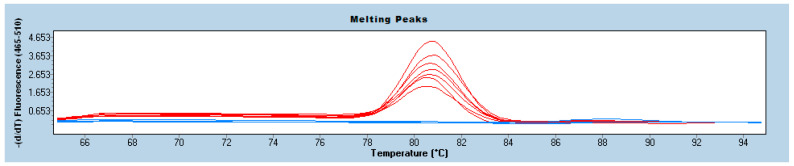
Melting curve graphs of some positive samples. Red: Positive samples, Blue: Negative samples.

The limit of detection (LOD) values of the LAMP reactions that were performed on the microcentrifuge tube and on the microfluidic device were 1.5 × 10^2^ CFU/mL and the limit of quantification (LOQ) values were 2.46 × 10^2^–7.4 × 10^2^ CFU/mL, respectively. The Cohen’s Kappa (κ) values of the LAMP reactions that were performed in the microcentrifuge tube and the microfluidic device were 0.620–0.705, respectively. Melting analysis and melting curve graphs were used to evaluate the LAMP reactions (Figure 5).

It was found that the sensitivity of the LAMP Reaction decreased following the application on the microfluidic device, but the data showed strong agreement with the Kappa Correlation Value according to the culture for the use of the microfluidic device in the detection of *S. pyogenes* (κ: 0.705) (Table 5).

## 4. Discussion

*S. pyogenes*, which is also known as Group A Streptococci (GAS), is an important pathogen and can cause serious infections in humans [1,2] with the highest incidence rate in children aged 5 to 15 [5]. Although the treatment with empirical penicillin is frequently used, the treatment failure rate has increased in recent years and reached 40% [7]. For this reason, early detection and identification of the pathogen is important [2]. Because of its different advantages, the LAMP method can form the basis of Point-of-Care (POC) diagnostic systems and can be used with unique designs for many pathogens [9]. Microfluidic devices, which are combined with molecular techniques such as LAMP, may provide an important option for the creation of rapid Point-of-Care diagnostic tests [10]. 3D printing systems are used increasingly in manufacturing because these devices work at low costs [14]. For all these reasons, we focused on creating a test device ready to detect *Streptococcus pyogenes* by applying the Loop-Mediated Isothermal Amplification (LAMP) method on a microfluidic test device via a 3D printer.

It is already known that LAMP design is performed by using the *S. pyogenes speB* gene and is used in clinical samples for rapid diagnosis [2,25,26]. In their study, Cao et al. reported that the sensitivity was 71.21% [2]. Henson et al. reported 100% sensitivity and 99.2% specificity [26]. In this study, it was found that the sensitivity of the LAMP Test, which was performed in a microcentrifuge tube with our LAMP design, was 92% and the specificity was 94%, which made us consider that this difference might have occurred because of the standard ATCC strains used in the study. Zhao et al. used a LAMP test by designing primers specific to the spy1258 gene of the *S. pyogenes* Serotype M1 SF370 strain. Similar to our study, they reported in their study that they created 10-fold dilutions between 14.86 μg/mL and 1.486 pg/mL, and the detection limit of the method was 1.486 pg, and this detection limit was 10-fold higher in PCR [27]. We did not obtain data on the detection limit versus PCR because we did not make a comparison with PCR or real-time PCR in our study. We have shown that the LAMP method is a nucleic acid amplification technique that can amplify the target DNA with high specificity and sensitivity in 30 min with a simple heater. The *speB* gene, located on the chromosome of the bacteria *S. pyogenes*, is a key factor in its ability to cause fever and damage the heart. All strains of *S. pyogenes* have this gene, and the protein it produces (*speB*) plays a crucial role in how the bacteria harm the body. *SpeB* helps *S. pyogenes* invade and infect human lung cells [28].

When the literature was reviewed for *S. pyogenes* detection with microfluidic device design, it was found that Huang et al. developed a microfluidic PCR array system for different respiratory tract infection agents, including *S. pyogenes*, and reported that it had 100% sensitivity [29], but this test differed from our study in that it was a very complicated system and could not be used in POC detection. Unlike our study, Mohajeri et al. developed a method for the diagnosis of *S. pyogenes* with nanoparticles and reported that they were able to detect it in 20 min [30]. It was found that there is no literature data on the detection of *S. pyogenes* in microfluidic devices designed with 3D printers. However, there are studies in which these methods were combined for the diagnosis of different pathogens and manufactured with 3D printers. These studies also show that the detection of different pathogens can be achieved by combining molecular methods on microfluidic device manufactured with 3D printers [31,32,33].

Current guidelines regarding the diagnosis of *S. pyogenes*-associated pharyngitis in children and adolescents recommend testing throat swabs with rapid antigen tests test-negative samples with culture and following rapid antigen detection [34]. Rapid antigen tests are easy to perform and have extremely high specificity and rapid return times that can be less than 15 min. However, the relatively low sensitivity of rapid antigen tests [70–90%] leads to the need for culture in rapid antigen test-negative samples in children and young adults. Bacterial culture remains the gold standard method with a high specificity [90–95%], but requires at least 24–48 h for a positive result to be detected [35].

Although the design of the present study is not the same, rapid antigen tests that are developed with immunochromatographic methods for the rapid diagnosis of *S. pyogenes* are widely used for diagnostic purposes [36,37,38]. In their study, Reijtman et al. attempted to develop an immunochromatographic method to detect *S. pyogenes* antigen directly from clinical samples and positive blood culture bottles. They reported the sensitivity of the test they developed as 97.1% and the specificity as 97.8% and argued that they obtained reliable results in less than 10 min [36]. GAS is known to be among the most common bacterial pathogens, especially in children who are older than 3 years, and the incidence of GAS infections has increased in invasive and non-invasive forms in recent years. GAS also frequently causes middle ear infections in children between the ages of 3 months and 15 years [37]. Cohen et al. conducted a multicenter immunochromatographic test trial in children who had middle ear infections and found that the performance of this test had a very high sensitivity [97.3%] and specificity [100%] when compared to the culture method. They also reported that diagnosis, especially made with rapid antigen tests, contributed to the prescription of narrow-spectrum antibiotics, which may reduce the development of antibiotic resistance [37]. Solvik et al. aimed to evaluate the diagnostic performance of two rapid antigen tests, the QuickVue Dipstick Strep A test and the DIAQUICK Strep A blue dipstick under real-life conditions. They reported the diagnostic sensitivity value of QuickVue as 92% and the diagnostic specificity value as 86%. They found the diagnostic sensitivity value to be 72% and the diagnostic specificity value 98% for DIAQUICK. They argued that although these tests were user-friendly, they were not good enough in terms of diagnostic performance [38]. It is already known that rapid diagnostic tests can provide faster results, especially in children, compared to the culture method and can help in deciding on antibiotic treatment. Although their sensitivity (ability to accurately detect the disease) and specificity (ability to accurately identify those without the disease) were reported to be high, test performances show great variability in studies due to unexplained reasons and must be supported by the culture method [39]. For this reason, nucleic acid amplification test (NAAT) methods have become popular with their fast return times and high sensitivity and specificity rates [35]. It was shown in different studies that especially molecular diagnostic tests provide better specificity and sensitivity compared to immunochromatographic methods [4,35,40]. In their study, Banerjee et al. aimed to detect the presence of Streptococcus pyogenes in throat swab samples with the real-time PCR method and developed the Revogene Strep A test. They reported in their multicenter study that the sensitivity and specificity of the Revogene Strep A test were 98.1% and 94.7%, respectively. They also showed that it had high sensitivity and specificity in detecting GAS and is a faster alternative for diagnosis [35]. In their study, Elf et al. developed a test by using the strand invasion-mediated amplification [SIBA] method, which is an alternative NAAT method to diagnose GAS, reporting that they could detect *S. pyogenes* DNA in amounts as low as ten copies within 13 min with this GAS SIBA method they developed. They also reported that the GAS SIBA method detected S. pyogenes from clinical samples with 100% sensitivity and specificity [4]. In this study, it was found that our detection limit was as low as 100 CFU/mL. However, the fact that our sensitivity and specificity were not that high made us considers that this might have occurred because of the method and microfluidic device we used.

Similar to the aim of our study, Rivas-Macho et al. aimed to design an electrochemical sensor based on Loop-Mediated Isothermal Amplification (LAMP) to detect *Listeria monocytogenes* and to create this device with a 3D printer. A set of 21 primers targeting the “*hly* gene” of *Listeria monocytogenes* was designed and one was selected to optimize the experimental design. The limit of detection has been reported to be 1.25 pg of genomic DNA. The device developed in this study was produced from transparent acrylic resin by using the SLA 3D printing technique, similar to our study, and showed that it can be used to detect pathogens [41]. Papadakis et al. developed a portable device to detect nucleic acids in raw samples rapidly and quantitatively, envisioning the device to be simple, inexpensive, and have a 3D-printed design. They tested the device in two different clinical applications. For the first clinical application, they performed cancer mutation analysis and pathogen detection such as a COVID-19 test for the second clinical application. The device was able to detect the BRAF V600E Mutation in cancer application successfully. When used for COVID-19 testing, it could detect SARS-CoV-2 RNA in both extracted and direct samples. Both clinical practices showed high sensitivity and specificity when compared to standard methods such as sanger sequencing, ddPCR, and qRT-PCR [42]. As an alternative approach to the diagnosis and treatment of multidrug-resistant bacteria, Glatzel et al. produced a disposable microfluidic device with 3D printing technology to produce, formulate, and test antibiotics in situ and found that the device was inexpensive, easy to use, and could be used by untrained staff. They reported that the microfluidic device, in this form, could be distributed on a global scale by using open-source software and hardware and could be an important approach in combating important pathogens such as multidrug-resistant microorganisms [43]. There is a study in the literature reporting that devices produced with 3D printing in this way can be used for the diagnosis of Xylella fastidiosa subsp. pauca [Xfp] (a bacterium infecting olive trees) after combining with the LAMP method. In this study, similar to our study, the data were compared with the culture method. The limit of detection was determined as 10^2^ CFU/mL and 169.2 target copies/µL. The LAMP method was also compared with different molecular analysis methods such as qPCR and ddPCR to detect Xfp in field samples. It was reported that the LAMP method showed 71% accuracy and 52.54% diagnostic sensitivity compared to qPCR, and 81.56% accuracy and 76.2% diagnostic sensitivity compared to ddPCR [44]. Zhou et al. developed LAMP reagents loaded into paper-based devices to detect foodborne pathogens such as *E. coli* O157:H7, *Salmonella* spp., and *S. aureus* in situ. For this system, they developed a smartphone-based portable device, a heating plate that provided accurate temperature control for isothermal amplification, an imaging area, and a chip interface that used 3D printing technology integrated with optical elements to collect fluorescent signals that are generated by the amplification reaction. As a result of this study, they showed that the device could be used successfully to detect multiple pathogens. They also emphasized that genetic diagnosis for food pathogens might have the potential to be used as a suitable screening method anywhere, anytime, and by everyone [45]. Kadimisetty et al. [11] also used a low-cost, 3D-printed microfluidic device for molecular diagnosis at the point of care. They used a different 3D printing methodology (SLA instead of FDM) and detected different pathogens (*Plasmodium falciparum* and *N. meningitidis*). This device also uses a photopolymerization technique to integrate membranes and reactors for nucleic acid isolation and amplification. The device also has a biocompatible coating for improved performance and can be used for both qualitative and quantitative detection of various targets. They also suggest that this technology has the potential to provide fast and reliable diagnoses in resource-limited settings [11]. FDM fabrication technique using in this study is a cheaper than SLA fabrication technique but all these studies show that different molecular diagnostic methods integrated with 3D printing can be used as screening methods that can be applied anytime, anywhere, and by anyone, as in our study. Combining these techniques seems to be applicable and developable in areas such as health, medicine, agriculture, and food. There is also an opportunity to improve the design for unique functionality towards PoC detection.

Although microfluidic chips produced using Fused Deposition Modeling (FDM) have a lower resolution compared to some other 3D printing methods, its advantages in terms of accessibility, cost, ease of use and rapid prototyping make it the choice for use [46,47]. Polylactic acid (PLA) is a sustainable and biocompatible material that has been investigated for use in microfluidic chip applications. The biocompatibility of the PLA (polylactic acid) material used in the study is a crucial aspect, especially when considering that the amplification process occurs within the chip. PLA is generally recognized as a biocompatible material, and its use in medical and biological applications, including tissue engineering and disposable devices [21]. Therefore, PLA material was selected for the design of the microfluidic device in our study and FDM method was used in 3D printing.

The limitations of this study were that clinical strains were not used in the study and that methods such as a colorimetric or electrochemical sensor were not integrated into the microfluidic device developed here. In particular, integrating additional methods such as a colorimetric or electrochemical sensor for the analysis of the results will contribute to the portability of the test and easier analysis of the results. Despite these limitations, the study is important in that it shows that microfluidic devices can be created with 3D printers, and can be combined with different molecular techniques to detect pathogens quickly with high specificity and high sensitivity.

## 5. Conclusions

The data obtained in the present study show that the LAMP method and microfluidic device technology can be combined to detect *S. pyogenes*, and these microfluidic devices can be manufactured by using 3D printers. Microfluidic devices developed by combining these techniques provide data close to laboratory equipment and tests and can be used for screening purposes. These devices can also be combined with LAMP reactions to detect different pathogens in areas where resources are limited and rapid results are needed. It can be applied and developed in different fields such as health, medicine, agriculture, and food. The resulting microfluidic devices will be able to provide low-cost and reliable diagnosis. However, large-scale studies must be conducted by combining the data with clinical isolates.

## Figures and Tables

**Table 1:** 3D printing parameters for microfluidic chip production.

Nozzle Diameter	Layer Thickness	Filling Rate	Nozzle Temperature	Heated Bed Temperature	Oppression Speed
0.4 mm	0.2 mm	100%	190 °C	60 °C	50 mm/s

**Table 2:** *S. pyogenes speB* gene-specific LAMP primer set.

Label	5′pos	3′pos	len	Sequence
F3	896	916	21	CTACCTACTTATAGCGGAAGA
B3	1089	1109	21	CTTCCCAATCTTGTTTGCTAA
FIP	917	993	48	GGACCATAATCCATGTCTACTGAAA-GAATCTAACGTTCAAAAAATGGC
BIP	1008	1087	41	CAGGTAGCTCTCGTGTTCAAAG-GTCGCTACGGTTAATTTGG
LF	948	968	21	AATTGATGGCTGATGTTGGTA
LB	1045	1064	20	CTTTGGCTACAACCAATCTG

**Table 3:** The strains used in the study and their intended use (×10 different repetitions were performed for each strain in the table).

Strain (CFU/mL)	Control Type
*S. pyogenes* ATCC 19615 (1.5 × 10^6^)	Positive Control
*S. pyogenes* ATCC 19615 (1.5 × 10^5^)	Positive Control
*S. pyogenes* ATCC 19615 (1.5 × 10^4^)	Positive Control
*S. pyogenes* ATCC 19615 (1.5 × 10^3^)	Positive Control
*S. pyogenes* ATCC 19615 (1.5 × 10^2^)	Positive Control
*Streptococcus agalactiae* ATCC 12386 (1.5 × 10^6^)	Negative Control
*Streptococcus mutans* ATCC 25175 (1.5 × 10^6^)	Negative Control
*Haemophilus influenzae* ATCC 49247 (1.5 × 10^6^)	Negative Control
*Staphylococcus epidermidis* ATCC 12228 (1.5 × 10^6^)	Negative Control
*Lactobacillus acidophilus* ATCC 4356 (1.5 × 10^6^)	Negative Control
*C. albicans* ATCC 10231 (1.5 × 10^6^)	Negative Control
*Klebsiella pneumoniae* ATCC 13883 (1.5 × 10^6^)	Negative Control
*Staphylococcus aureus* ATCC 29213 (1.5 × 10^6^)	Negative Control
*Escherichia coli* ATCC 25922 (1.5 × 10^6^)	Negative Control
*Pseudomonas aeruginosa* ATCC 27853 (1.5 × 10^6^)	Negative Control

**Table 4:** (**a**) The results of the *S. pyogenes* LAMP method on the microfluidic device according to the culture method. (**b**) The results of the *S. pyogenes* LAMP method on the microcentrifuge tube according to the culture method.

**(a)**				
***S. pyogenes***		**Culture**		
		**Positive**	**Negative**	
LAMP rxn on the microfluidic device	Positive	42	10	52
	Negative	8	90	98
		50	100	
**(b)**				
***S. pyogenes***		**Culture**		
		**Positive**	**Negative**	
LAMP rxn on microcentrifuge tube	Positive	46	6	52
	Negative	4	94	98
		50	100	

**Table 5:** The distribution of the analytical data of LAMP reactions that were performed on the microcentrifuge tube and on the microfluidic device.

Target	Test Performance Indicator	LAMP Reaction Performed on the Microfluidic Device	LAMP Reaction Performed on the Microcentrifuge Tube
*S. pyogenes*	Sensitivity	84.00%	92.00%
	Specificity	90.00%	94.00%
	Positive Predictive Value (PPV)	80.77%	88.46%
	Negative Predictive Value (NPV)	91.84%	95.92%
	Limit of Detection (LOD)	1.5 × 10^2^	1.5 × 10^2^
	Limit of Quantification (LOQ)	7.4 × 10^2^	2.46 × 10^2^
	Cohen’s Kappa (κ) value	0.705	0.620

## Data Availability

Data are contained within the article.
